# Functional Neurological Disorders as Seen by a Cohort of General Practitioners in Northern Italy: Evidence From an Online Survey

**DOI:** 10.3389/fneur.2021.583672

**Published:** 2021-01-25

**Authors:** Angela Marotta, Mirta Fiorio, Isabella Fracasso, Carlo Andrea Franchini, Giovanni Defazio, Michele Tinazzi

**Affiliations:** ^1^Department of Neurosciences, Biomedicine and Movement Sciences, Università di Verona, Verona, Italy; ^2^Italian Society of General Practice and Primary Care (SIMG)–Section of Verona, Verona, Italy; ^3^Neurology Unit, Department of Medical Sciences and Public Health, University of Cagliari and AOU Cagliari, Cagliari, Italy

**Keywords:** functional neurological disorders, conversion disorders, general practice, education, survey

## Abstract

General practitioners (GPs) provide primary care and advise their patients on which diagnostic and therapeutic pathways they judge most appropriate. For patients with functional neurological disorders (FND), receiving a proper explanation of diagnosis by their GP from the very beginning may drastically improve prognosis. Novel approaches to the diagnosis and treatment of FND have important implications for effective management. The aim of this study was to investigate Italian GP opinion and knowledge about FND in light of new approaches to the illness. To do this, we evaluated the responses to a 13-item web-based survey completed by 133 GPs practicing in northern Italy. Psychological terms to describe FND were more frequently used than functional neurological disorder and mental illness was considered an important predictor of diagnosis. Referral to a neurologist rather than to a psychiatrist was largely preferred, while physiotherapy consultation was seldom recognized as a valuable approach to treating FND. Overall, the survey findings suggest that knowledge about novel approaches to FND is somewhat lacking. Currently, GPs appear to be transitioning from a classical psychological view of the disorder toward a more modern conceptualization, in which neurobiological, psychological, and social factors all play an important role. Professional education during this transition would be an advantageous way to optimize physician management of FND and to enhance diagnosis, explanation, and management across primary and secondary care pathways.

## Introduction

General practitioners (GPs) often encounter patients presenting with symptoms unexplained by organic damage (1). In patients with functional neurological disorders (FND) for example, symptoms are characterized by clinical signs outside the normal rules of neurological disease (2). Common presentations are functional movement disorders and dissociative seizures. The functional movement disorders present with motor symptoms (e.g., tremor, dystonia, gait abnormalities) that may disappear or subside with distraction. Dissociative seizures resemble epileptic seizures or syncope without abnormal electrical brain activity (3). Due to unclear diagnosis and management, patients with FND may receive the wrong diagnosis or a poor explanation of their medical condition, resulting in misunderstanding and leading to inappropriate treatment and poor outcome (4).

Our understanding of FND has made considerable advances, with implications for diagnosis and treatment. Research into the pathophysiology of FND has challenged old assumptions about the disease as a primarily psychological illness (5). It has been widely demonstrated that symptoms are more consistently explained by abnormalities in high-order cognitive functions involving attention and sense of agency (e.g., feeling of control over abnormal bodily movement) rather than by psychological difficulties (6). The development of novel approaches to the diagnosis and treatment of FND has gone forward with this modern conceptualization of the disease. Positive clinical signs have been clearly identified that distinguish functional neurological symptoms from their organic counterpart (e.g., functional tremor but not organic tremor is modified by distractive maneuvers) (4). Recent FND management guidelines recommend a multidisciplinary stepped approach in which care is delivered by a coordinated specialist team (e.g., neurologist, psychiatrist, physiotherapist, psychotherapist) (7). In addition, a growing body of evidence suggests that such approaches improve the efficacy of managing FND (4, 8–10).

Nonetheless, many health care professionals, including GPs, find FND difficult to manage (3, 11). Studies across different countries investigating the opinions and clinical experience of health care providers [e.g., (3, 12–18)] have revealed that poor knowledge about novel approaches to FND diagnosis and treatment might explain at least in part the difficulties in dealing with FND. The studies involved mainly specialists (e.g., neurologists, psychiatrists, psychologists, physiotherapist) and left almost completely unexplored the opinions and clinical experience of GPs - the primary providers of health care.

Exploring GP attitudes toward FND deserves particular attention since they play a pivotal role in health care (7). They are the initial contact with the national health system. As such, they act as gatekeepers to accessing most secondary and specialist care, besides providing patients with a clear explanation of their symptoms from the very beginning. Moreover, because they are familiar with their patients' medical history, they can consult with specialists for making informed decisions. Finally, they can support management of the disease by ensuring continuity and coordination of care in the community (19) and help patients understand the diagnosis, thus increasing the chances for adherence to therapy and for favorable outcome (20). Despite their potential role in improving primary care for FND, little is known about the opinions and clinical experiences of GPs regarding FND.

With this exploratory study we investigated the opinion, knowledge, and clinical experience of a cohort of Italian GPs regarding FND. To do this, we developed an *ad hoc* online survey. The items were focused on areas (terminology, diagnosis, and management strategies) that have been consistently reworked in light of the novel approach to FND. We also explored GP attitudes toward FND, their role in diagnosis and treatment, and their interest in improving their understanding of the disease.

## Materials and Methods

The findings of this *ad hoc* quantitative exploratory web-based cross-sectional survey are reported according to the Checklist for Reporting Results of Internet E-Surveys (CHERRIES) guidelines (21) and to STrengthening the Reporting of OBservational Studies in Epidemiology (STROBE) (22). An initial literature review was conducted to assess previous studies examining the opinion, knowledge, and clinical experience of health care professionals with medical conditions that are not explained by organic damage. Based on these studies (3, 12, 23, 24), we created a preliminary questionnaire, which was then modified according to the feedback from two GPs (CAF, IF). The final 13-item questionnaire version is divided into three sections as described below.

*Section 1: Demographic and professional characteristics*. Four items investigated demographic (age, sex) and professional characteristics (number of years practiced as GP and number of patients).

*Section 2: Opinion, knowledge, and clinical experience*. The first of seven items investigated exposure to FND (i.e., number of patients that GPs believe have symptoms not explained by an organic cause). The second was a multiple-choice question about the terms used to name FND. The third focused on predictors of diagnosis. The respondents rated the extent to which they felt certain criteria were predictive for a diagnosis of FND as “not predictive at all,” “not very predictive,” “somewhat predictive,” “very predictive,” “extremely predictive,” or “I don't know.” The fourth item investigated opinions about the adequacy of specialist consultation/treatment. The respondents rated the degree of adequacy (i.e., “not adequate at all”, “not very adequate”, “partly adequate”, “very adequate”, or “highly adequate,” and “I don't know”) of four specialist consultations and five types of treatment for FND. The fifth regarded the management strategies the respondents used to deal with their patients. The respondents stated the extent to which they agreed (“strongly disagree,” “disagree,” “uncertain,” “agree,” “strongly agree”) with options for initial intervention in FND. The sixth item was a multiple-choice question on the role of GPs in FND diagnosis and treatment. The final item investigated the level of satisfaction on an 11-point scale from 0 (no satisfaction) to 10 (high satisfaction) in dealing with patients with FND. The items in this section are reported in Supplementary Table 1.

*Section 3: Interest in FND*. The third section consisted of two dichotomic items investigating interest in FND.

### Data Collection

GPs were recruited from the members of two Italian GP societies: the Italian Society of General Practice and Primary Care (SIMG) – Section of Verona, and the Federazione Italiana Medici di Medicina Generale (FIMMG). Only society members practicing in Verona were invited to take part at the survey. The Google Forms online tool was used (Google LLC, Menlo Park, CA, USA). Data were collected over a 18-weeks period (30 October 2017–7 March 2018). An e-mail from the Verona chapter of SIMG (SIMG -Verona) inviting participation in the study was sent to the 397 SIMG and FIMMG members practicing in Verona. The e-mail explained that the study investigated GP opinions, knowledge, and clinical experience with *non-organic* neurological disorders. The neutral term *non organic* was used to avoid connotations with presumed pathophysiological mechanisms underlying the disease (i.e., “functional” or “psychological”). An example of what we meant by a “non-organic” neurological disorder (neurological symptoms, like tremor, that may disappear with distraction) was also given to exclude potential bias due to misleading terminology. The invitation contained the survey link, and personal data handling (anonymity). Also, GPs were informed that the survey took about 5 min to complete. Four e-mail reminders were sent by SIMG at 2, 4, 8, 12, and 16 weeks after the initial mailing. Response to all survey items was mandatory. If a response to an item was missed, an alert appeared on the screen and the respondent had to respond to the item in order to proceed to the next one. The study was approved by the ethics committee of the University of Verona and was conducted in accordance with the Declaration of Helsinki. Response to the survey was assumed to grant consent (23).

### Data Analyses

Data were downloaded from the Google Forms onto Excel sheets and reviewed for accuracy. Analysis was performed using SPSS software (version 19). Responses were analyzed with descriptive statistics, expressed as the mean (±standard deviation [SD]) and percentage for continuous and categorical variables, respectively. Data from Section 2 were analyzed in depth after converting responses to predictors of diagnosis, specialist consultation, treatment adequacy for FND, and management strategies into a 5-point Likert scale from 1 (“not predictive at all,” “not adequate at all,” and “strongly disagree”) to 5 (“extremely predictive,” “extremely adequate,” and “strongly agree”). The distribution average of the responses to each item (i.e., predictors of diagnosis, perceived usefulness of consultations/treatments, GP management strategies) was compared using the Wilcoxon signed-rank test. Spearman's correlations were performed to investigate potential correlations between respondent age and years of medical practice. The difference in the number of male and female GPs was analyzed using the chi-squared test. Statistical significance was set at *p* < 0.05.

## Results

### Section 1: Demographics and Professional Characteristics

The overall response rate was 39% (*n* = 133). Most respondents were men (*n* = 82, 62%) (chi-squared 7.22, *p* = 0.007) and >51 years old (*n* = 104, 78%) (mean age ± SD, 57 ± 9.01). The majority had been practicing for more than 20 years (*n* = 88, 66%) (mean years practiced, 24.02 ± 12.48), with a catchment area of more than 1,000 patients (*n* = 121, 91%) (Table 1).

**Table 1:** Demographic and professional characteristics.

**Sex**	**Responses–no. (%)**
Male	82 (62)
Female	51 (38)
**Age (years)**	
<40	9 (7)
41–50	20 (15)
51–60	40 (30)
>60	64 (48)
**Years of practice**	
<10	25 (19)
11–20	19 (14)
21–30	37 (28)
>30	51(38)
**Patients registered in the practice (total)**	
<=500	2 (1)
501–1000	10 (8)
>1000	121 (91)

### Section 2: Opinion, Knowledge, and Clinical Experience

#### Exposure to FND

For the item “In your opinion, how many of your patients might present with neurological symptoms without an organic cause?” the majority (*n* = 78, 59%) reported they believe that <10% of their patients might present neurological symptoms without an organic cause. Many (*n* = 42, 32%) estimated a higher proportion of these disorders among their patients (10–25%). Few (*n* = 8, 6%) believed that 25–50% of their patients were likely to be affected by FND, while none reported that more than half of their patients might have FND. Very few were unable to estimate how many of their patients might have FND (*n* = 5, 4%) (Table 2).

**Table 2:** Exposure to patients with FND and terms chosen to define the disease.

**Patients with FND registered in the practice**	**Responses–no. (%)**
<10%	78 (59)
10–25%	42 (31)
25–50%	8 (6)
>50%	0 (0)
I don't know	5 (4)
Terminology*	
Functional neurological disorders	71 (27)
Somatoform disorders	58 (23)
Unspecific anxiety syndrome	31 (12)
Stress-related syndrome	24 (9)
Depression	22 (8)
Non-organic disorder	21 (8)
Psychogenic disorder	19 (7)
Conversion disorder	5 (2)
Medically unexplained disorder	2 (1)
Hysteria	0 (0)

GP, general practitioner; FND, functional neurological disorders; *More than one response allowed.

#### Terminology

Respondents could choose from a list of 10 terms they usually used to describe neurological symptoms without an organic cause. More than one response was allowed for this item. A total of 259 responses were collected, including the free-text responses (*n* = 6), with the majority of respondents (*n* = 79, 60%) selecting more than one response. “Functional neurological disorders” (*n* = 71/259, 27%) and “Somatization disorder” (*n* = 58/259, 23%) were the two most frequent responses (Table 2). The term “Functional neurological disorders” was selected 30 times alone and 41 times together with “Conversion disorder” (*n* = 3), “Somatization disorders” (*n* = 21), “Depression” (*n* = 6), “Unspecific anxious syndrome” (*n* = 9), “Psychogenic disorder” (*n* = 9), “Medically unexplained syndrome” (*n* = 1), “Non organic neurological disorder” (*n* = 12). The term “Somatization disorder” was selected 9 times alone and 49 times together with “Functional neurological disorders” (*n* = 21), “Conversion disorder” (*n* = 3), “Depression” (*n* = 15), “Unspecific anxious syndrome” (*n* = 21), “Stress related disorder” (*n* = 20), “Psychogenic disorder” (*n* = 8), and “Non organic neurological disorder” (*n* = 5).

**Figure 1:**
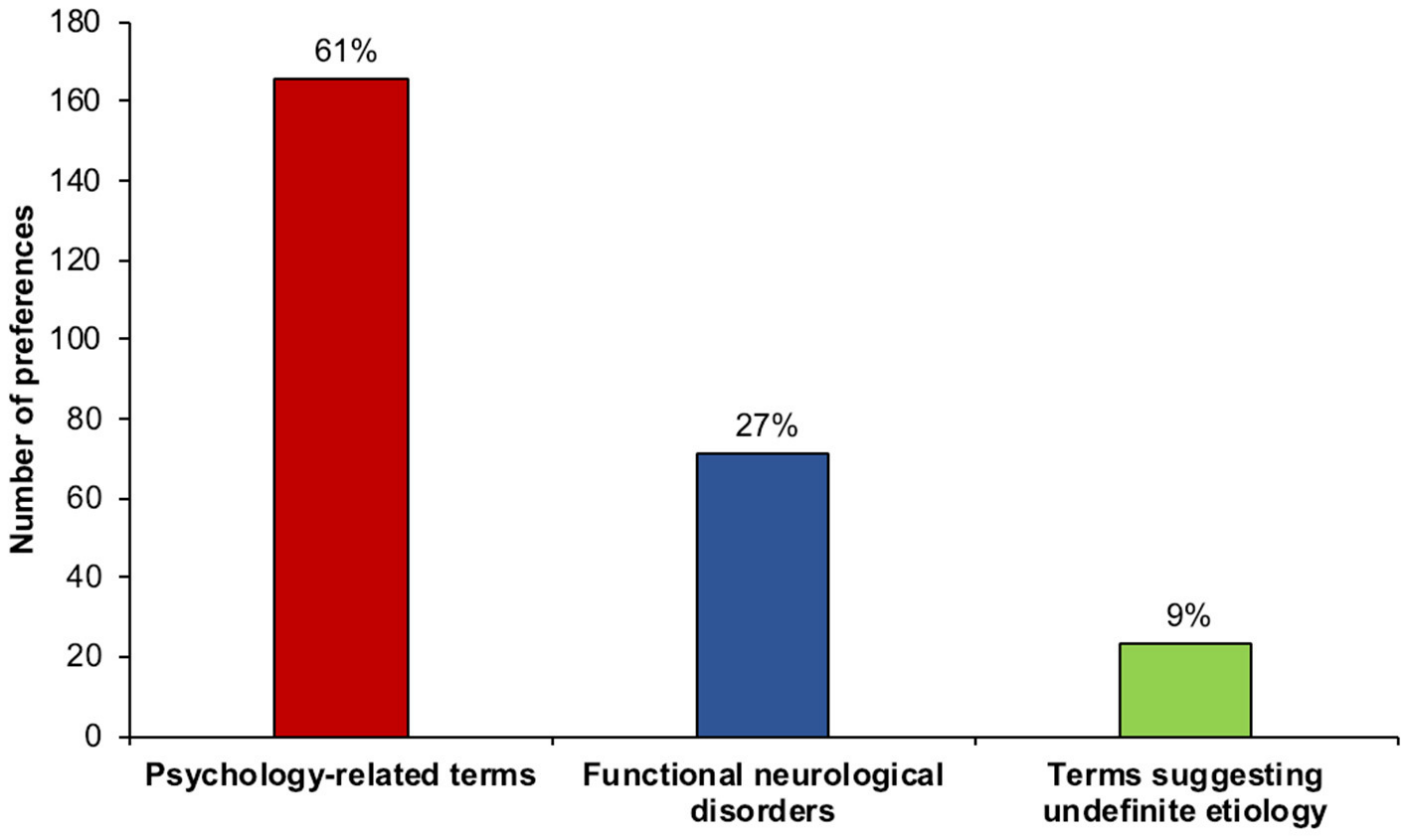
Number and percentage of preference for terms used to refer to symptoms not explained by organic damage. The group of psychology-related terms received more responses than the more appropriate term functional neurological disorder, while the terms suggesting an undefined etiology were least often selected.

Overall, psychology-related terms (e.g., “Somatization disorder,” “Unspecific anxious syndrome,” “Stress-related syndrome,” “Psychogenic disorder,” “Conversion disorder”) were selected more often (*n* = 159/259, 61%) than the novel term “Functional neurological disorders” (*n* = 71/259, 27%), indicating that a psychological view of FND is still widely held by this sample of GPs. Terms related to uncertain etiology (“Non-organic disorders” and “Medically unexplained disorders”) were less often selected (*n* = 23/259, 9%) (Figure 1), suggesting that indefinite definitions of FND were generally unacceptable.

Finally, free-text responses were included to capture any other terms in use; for example: symptom description (*n* = 2), intentional tremor (*n* = 1), fibromyalgia (*n* = 1), neurological disease under assessment (*n* = 1), cognitive deficit due to side effect of statins in patients with genetic predisposition (*n* = 1).

#### Predictors of Diagnosis

**Figure 2:**
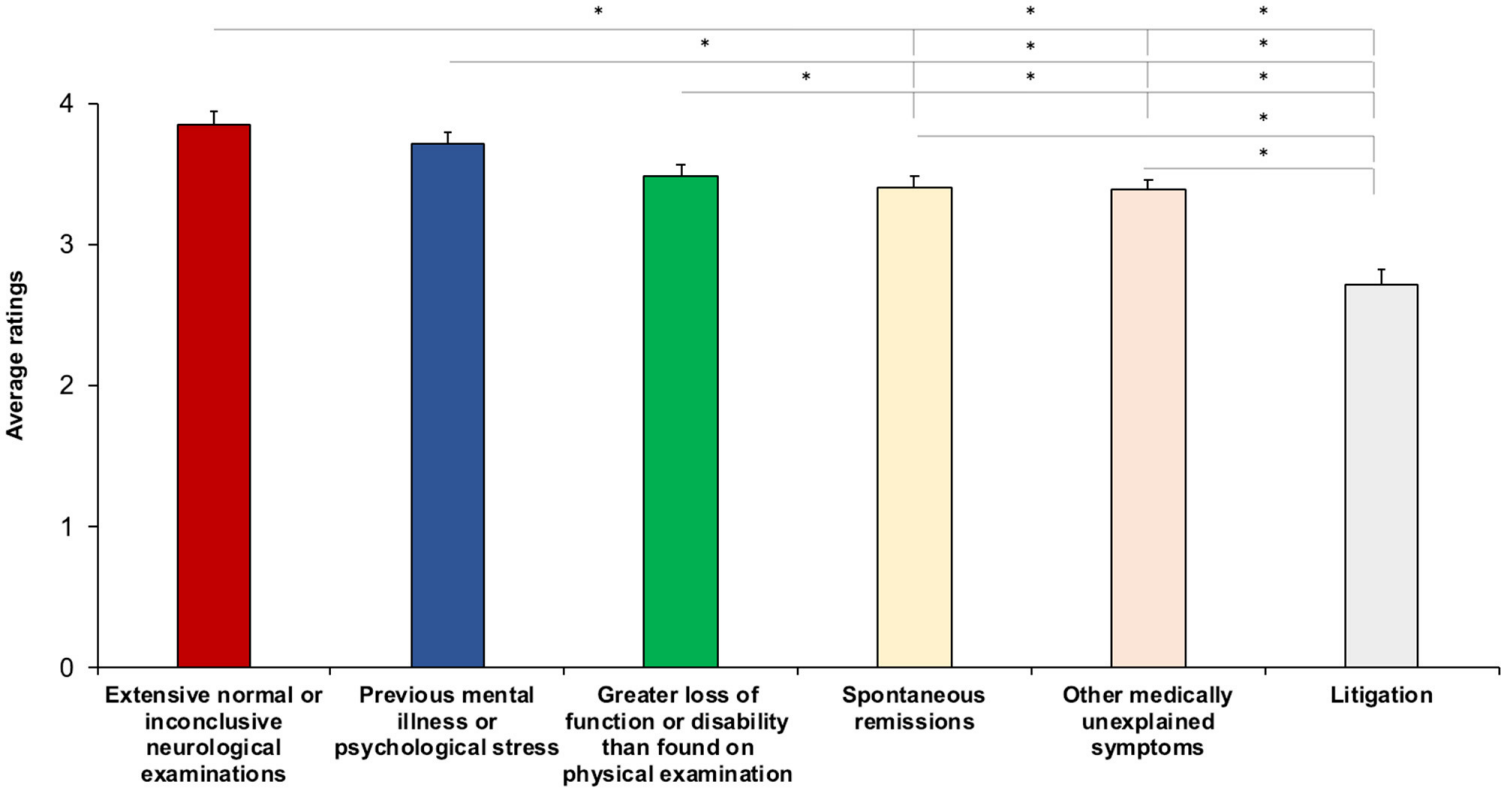
Average rating for predictors of the diagnosis of FND. The higher the score, the higher the degree of predictivity. Asterisks show significant differences. Error bars represent standard errors.

When asked to judge the extent to which certain diagnostic criteria were predictive for FND (from “not predictive at all” to “extremely predictive”) (Supplementary Table 2), on average, respondents rated “Extensive normal or inconclusive neurological examination” (3.86 ± 1.03), “Previous mental illness or psychological stress” (3.71 ± 0.98), and “Excessive loss of function or disability relative to examination findings” (3.48 ± 0.92) higher than “Spontaneous remissions” (3.40 ± 0.97) and “Other medically unexplained symptoms” (3.38 ± 0.82) (all *p* < 0.01). “Litigation” was rated lowest among all options (2.72 ± 1.15) (all, *p* < 0.001): either “not predictive at all” (*n* = 23) or “not very predictive” (*n* = 21) (Figure 2). The degree of predictivity of the diagnostic criteria did not correlate with age (all, *p* > 0.09) or years of practice (all, *p* > 0.10).

#### Specialist Consultation

**Figure 3:**
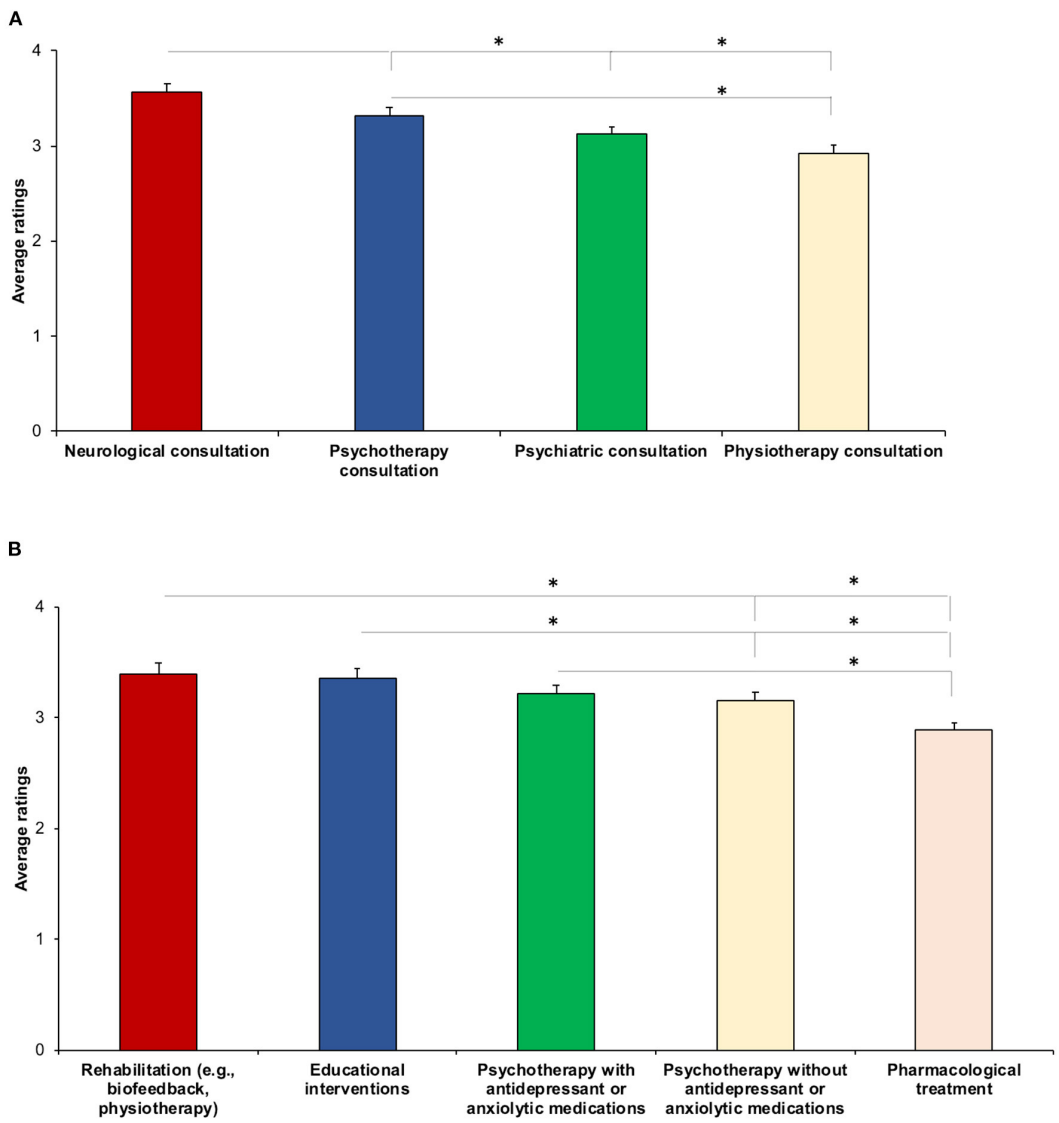
Average ratings for specialist consultation **(A)** and treatment **(B)**. The higher the score, the higher the specialist consultation/treatment was rated adequate for FND. Asterisks show significant differences. Error bars represent standard errors.

When asked to judge the extent to which certain specialist consultations were adequate for FND (from “not adequate at all” to “extremely adequate”) (Supplementary Table 3), the respondents rated “Neurological consultation” (3.57 ± 0.97) highest among all options (all *p* < 0.038). “Psychotherapy consultation” (3.31 ± 1.03) was rated higher than “Physiotherapy consultation” (2.92 ± 1.00) (*Z* = −3.26, *p* = 0.01). “Psychiatric consultation” (3.12 ± 0.96) was rated equally adequate for FND as both “Psychotherapy consultation” (*Z* = −1.85, *p* = 0.06) and “Physiotherapy consultation” (*Z* = −1.46, *p* = 0.14) (Figure 3A). These results did not correlate with age (all, *p* > 0.30) or years of practice (*p* > 0.21).

#### Treatment

When the GPs were asked to indicate the degree of adequacy of five different treatments for FND (from “not adequate at all” to “extremely adequate”) (Supplementary Table 3), on average, their responses did not statistically differ for degree of adequacy of “Rehabilitation (e.g., biofeedback, physiotherapy)” (3.40 ± 0.99), “Educational interventions” (3.36 ± 0.99), and “Psychotherapy with antidepressant or anxiolytic medications” (3.22 ± 0.91) (all *p* > 0.07), suggesting that they believed them to be equally adequate for treating FND. Moreover, “Psychotherapy without antidepressant or anxiolytic medications” (3.16 ± 0.80) was rated lower than “Rehabilitation” (*Z* = −2.42, *p* = 0.02) and “Educational interventions” (*Z* = −2.07, *p* = 0.04). Finally, “Pharmacological treatment” (2.89 ± 0.79) was rated lowest among all treatments (all *p* < 0.03), indicating that the pharmacological approach was judged the least adequate for treating FND (Figure 3B). Age significantly correlated with the degree of adequacy of “Psychotherapy without antidepressant or anxiolytic medication” for FND (*r* = −0.19, *p* = 0.03), suggesting that the younger the respondent, the higher that psychotherapy without pharmacological treatment was judged adequate for treating FND. Significant correlations were also found between years of practice and two treatments: “Psychotherapy without antidepressant or anxiolytic medication” (*r* = −0.21, *p* = 0.02) and “Rehabilitation services” (*r* = −0.21, *p* = 0.02), indicating that the fewer the years of practice, the higher the adequacy of these two treatments was rated.

#### Management Strategies

**Figure 4:**
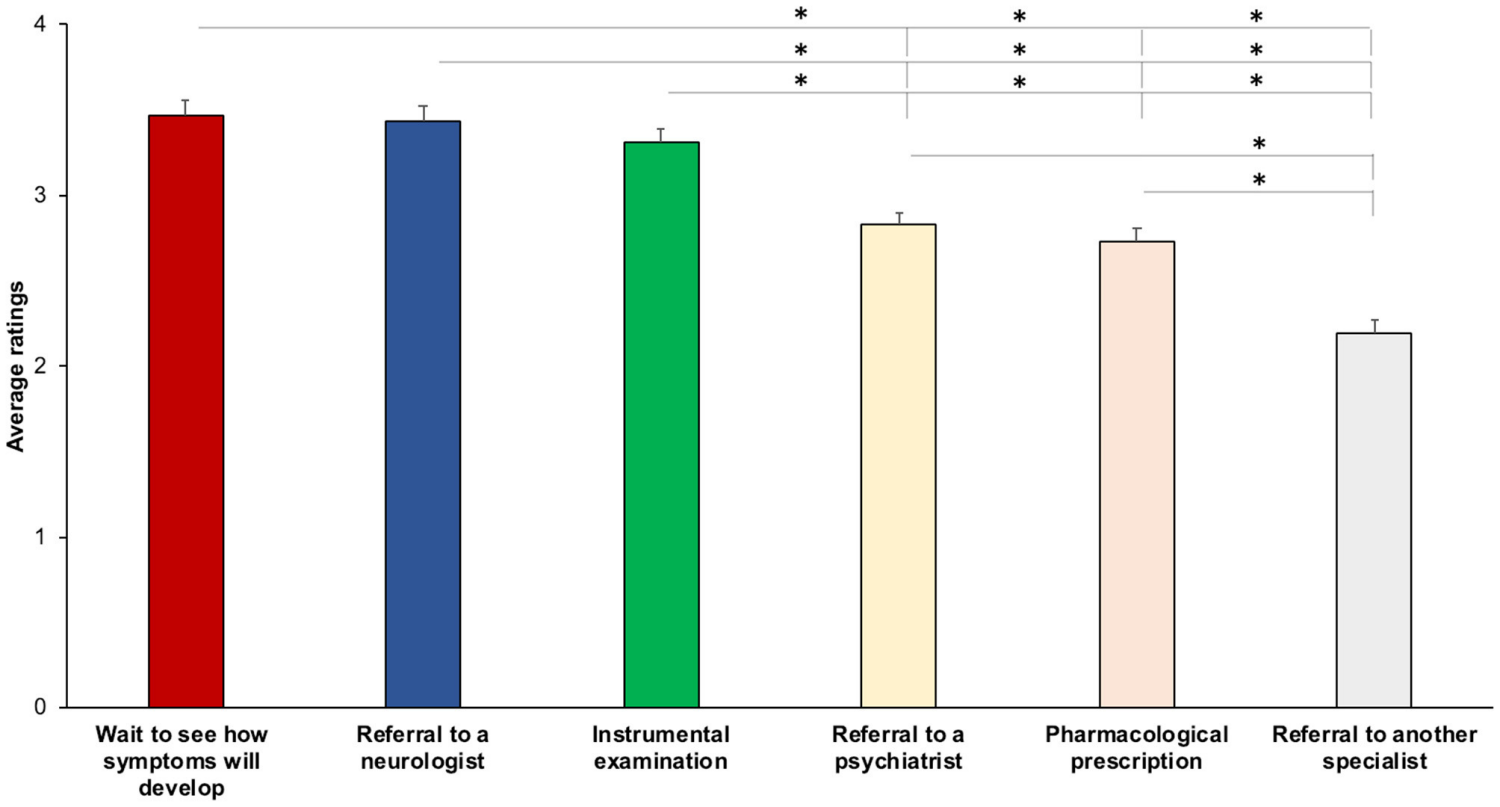
Average ratings for management strategies as the first intervention. The higher the score, the higher the agreement with the strategy. Asterisks show significant differences. Error bars represent standard errors.

When asked to indicate their level of agreement (from “extremely disagree” to “extremely agree”) with management strategies that they would adopt as a first step when dealing with FND (Supplementary Table 4), the respondents were equally oriented toward: “Wait to see how symptoms develop” (3.47 ± 1.00), “Referral to a neurologist” (3.44 ± 1.05), and “Instrumental examination” (3.31 ± 0.95) (all, *p* > 0.208). These interventions were more often selected than “Referral to a psychiatrist (2.83 ± 0.96), “Pharmacological prescription” (2.73 ± 0.87), and “Referral to another specialist” (2.19 ± 0.90) (all *p* < 0.001). “Referral to a psychiatrist” and “Pharmacological prescription” (*p* = 0.435) were rated higher than “Referral to another specialist” (all *p* < 0.001) (Figure 4). The degree of agreement with the management strategies did not correlate with age (all, *r* < 0.08, *p* > 0.188) or years of practice (all, *r* < 0.09, *p* > 0.283).

#### Satisfaction

When asked to rate the degree of satisfaction in managing FND on an 11-point scale from 0 (not at all) to 10 (extremely satisfied), on average, the respondents stated they were satisfied with how they dealt with FND (5.48 ± 2.16). The majority (*n* = 93, 70%) rated their level of satisfaction between 5 and 10. The satisfaction level did not correlate with age (*r* = −0.09, *p* = 0.28) and years of practice (*r* = −0.12, *p* = 0.15).

#### The Role of GPs

**Figure 5:**
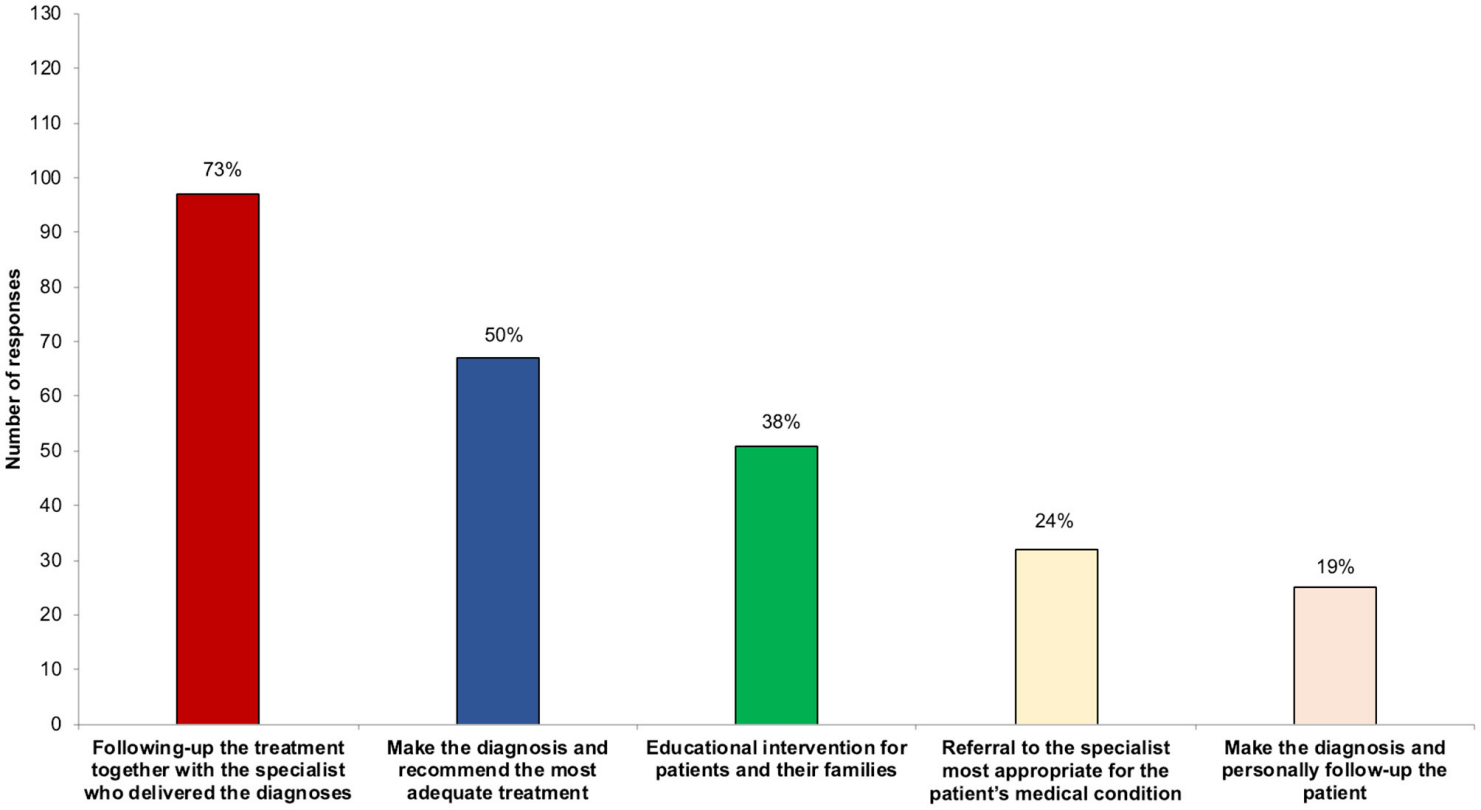
Number and percentage of how GPs see their role in caring for patients with FND.

When asked about their role in the management of FND, the majority (*n* = 94, 71%) gave more than one response, with “Following-up the treatment together with the specialist who delivered the diagnoses” (*n* = 97, 73%) the most frequent one; “Make the diagnosis and recommend the most adequate treatment” (*n* = 67, 50%) and “Educational intervention for patients and their families” (*n* = 51, 38%) were also frequently given. “Referral to the specialist most appropriate for the patient's medical condition” was selected by 32 GPs (24%). Finally, some believed that their role was “Make the diagnosis and personally follow-up the patient” (*n* = 25, 19%)” (Figure 5).

### Section 3: Interest in FND

The majority in our sample were interested in improving their knowledge of FND through access to more information (*n* = 119, 89%) or by taking part in professional courses on these disorders (*n* = 108, 81%).

## Discussion

FND are associated with long-term disability, poor quality of life, and extensive health care utilization with a huge economic impact on the health care system (25). GPs can do their part to improve care for patients with FND by referring them to the most appropriate diagnostic and therapeutic pathways from the very beginning. Yet little is known about their actual knowledge of the novel approaches to FND diagnosis and treatment. With this exploratory study we provide a picture of the opinions, knowledge, and clinical experience of a sample of Italian GPs concerning FND in light of recent advances.

Our main findings suggest that a clear idea of FND is lacking among GPs. Their opinions and knowledge appear to be in a sort of limbo between a modern approach to the disorders and outdated psychological models.

### Opinions and Knowledge About FND

That the current approach to FND encompasses both modern and classical (psychological) views of these disorders clearly emerged when the GPs were asked about the terms they usually adopt for symptoms without an organic cause. Use of terminology reflects the many different ways of conceptualizing and approaching the problem of patients with symptoms that are unexplained by the disease (26). In our sample, the terms “non-organic disorder” and “medically unexplained disorders” were among the least used. They often convey diagnostic uncertainty and the need to continue investigating for other causes (2). The low rating for these terms is likely suggestive of the GPs' need for a clear explanation of the disease, with implications for a potential reduction of unnecessary and costly diagnostic testing. Nonetheless, a definite conceptualization of FND seems to be missing, as suggested by the fact that the majority selected more than one term to describe symptoms without an organic cause, among which “Functional neurological disorder” was the most frequently selected. This suggests that the GPs were more likely to describe the cause of FND as a change in nervous system function rather than structure (26). Psychology-related terms (e.g., “Somatization disorders,” “Unspecific anxiety syndrome”) were more frequently selected than “Functional neurological disorder.” Previous studies demonstrated that patients with FND often feel disbelieved or misunderstood about their condition when health care professionals explain the disorder in terms of psychological difficulties, which undermines patient compliance (27). Conversely, the term “Functional neurological disorders” is preferred by both patients and health professionals since it is neutral with respect to the etiology of the disease, refers to the way in which symptoms manifest, increases a patient's understanding and acceptance of diagnosis, and reduces the fear of social stigma (2, 26–28). The low rate of response to “Functional neurological disorder” may stem from less familiarity with this term compared to psychology-related terms. Otherwise, the terminology usage in our sample suggests that while a modern view of FND is emerging, the classical psychological conceptualization of the disorder is still common among GPs.

This emerged also from responses to the items about predictors of diagnosis, specialist consultations, treatment, and management strategies. Although the term “psychological stressors” has been removed from the diagnostic criteria of the Diagnostic and Statistical Manual of Mental Disorders (DSM-5), previous mental illness or psychological stress was rated among the most predictive criteria and often ranked as “very predictive” or “extremely predictive.” This shows that many still hold a psychological view of FND and lack knowledge about new diagnostic criteria. Another observation is that litigation was rated as the least predictive factor for a diagnosis of FND. Since litigation has long been linked to feigned symptoms or malingering, our results suggest that the respondents felt patient-reported symptoms to be generally genuine and not faked to obtain secondary gain, with implications also for forensic medicine.

According to the most recent guidelines for FND diagnosis (4), “Neurological consultation” was ranked as the most adequate for FND. More respondents stated they would refer a patient to a neurologist than to a psychiatrist as a first intervention; this may be interpreted as less consideration given to psychiatric connotations of the disorder and closer alignment with the neurological approach. Moreover, referral to a psychiatrist would require the GPs to talk with their patient about a possible association between symptoms and psychiatric or psychological problems. One interpretation is that GPs have little familiarity with discussing psychological issues with their patients, thus avoiding referral to a psychiatrist in the first instance. Indeed, previous studies reported that many health professionals, including GPs, often struggle with discussing mental health problems (3). Another observation is that about 50% of the respondents selected instrumental examination as the first management strategy, suggesting the concern to exclude potential neurological damage. This might be related to the GPs' fear of missing an underlying pathological condition related to structural damage (1). Notably, however, neurological consultation is often sufficient *per se* to distinguish functional symptoms from their organic counterpart (29), without the need for diagnostic testing. Promoting knowledge about how neurological assessment can provide sufficient evidence for FND diagnosis might reassure GPs, while reducing costly and unnecessary testing.

Psychotherapy was selected as one of the most adequate treatments for FND, together with educational intervention and rehabilitation (e.g., biofeedback, physiotherapy). Recent evidence has shown the effectiveness of psychological therapy (e.g., cognitive behavioral therapy) for seizure reduction, improved quality of life, and better global functioning in patients with dissociative seizures (30). Moreover, improved outcomes have also been found with educational intervention providing a clear explanation of the diagnosis (10). Finally, significant improvements in functional movement disorders have been found after multidisciplinary rehabilitation (9) as well as physiotherapy (31–33). The results about physiotherapy in our sample deserve special mention. Although rehabilitation, including physiotherapy, was rated among the most adequate treatments, physiotherapy consultation was considered the least adequate for treating FND. These contrasting findings might suggest that although GPs recognize an important role for rehabilitation services in the treatment of FND, they probably lack information about the exact contribution that physiotherapy makes to management of the disease. Respondent age and years of practice correlated negatively with psychotherapy and rehabilitation treatments. These correlations indicate that younger age and fewer years of medical practice were associated with recognizing the adequacy of these interventions. This might be suggestive of more updated education about FND among younger GPs.

Remarkable findings also regarded the pharmacological approach to FND, which was considered less useful compared to rehabilitation and psychotherapy. Accordingly, pharmacological prescription was rarely endorsed by the GPs as the first approach to the disorder, suggesting their awareness that drug therapy is not particularly effective.

### Attitudes Toward FND

Our findings highlight that the respondents were, on average, generally pleased with how they managed patients with FND. The majority indicated a high level of satisfaction when dealing with these disorders. This contrasts with evidence from previous surveys that found negative attitudes toward FNDs among specialists (e.g., neurologists, psychiatrists, nurses, physiotherapists) (3, 6, 34). Moreover, the majority of our respondents had multiple roles in managing their patients with FND. A collaborative intervention based on cooperation between GPs and other health care professionals was largely preferred by our sample. This is in line with a stepped care approach that involves diverse health professionals and is highly recommended for managing patients with FND (7). Furthermore, providing educational support to patients and their family was also frequently indicated as a potential role for GPs in the management of FND. This is encouraging, since an adequate explanation of the diagnosis, reassurance, and education are fundamental for the treatment of FND (29). Overall, our results show that the GPs had a positive attitude toward FND, which is a requisite for effective intervention. Nonetheless, the majority expressed a need for further education about FND, likely suggesting a perceived lack of sufficient knowledge to deal with FND, as reported by previous surveys involving other health professionals (3, 18, 34).

### Strength and Limitations

The study findings provide a picture of the current knowledge base, opinions, and clinical experience of a sample of Italian GPs about novel approaches to FND. Several limitations need to be acknowledged. The study sample was small, limiting the generalizability of our findings. Nonetheless, the response rate was in line with previous studies (3, 12, 24) and the demographic and professional characteristics of the sample are representative of the Italian GP population (35). The majority were men over age 50, with more than 20 years of practice and a list of more than 1,000 patients. Another limitation is that we did not include a question about confirmed diagnoses of FND, which limits the interpretation of our findings. In the context, the estimate of exposure to FND might have been biased by uncorrected diagnosis (e.g., organic disorders diagnosed as non-organic disorders). However, our findings are in line with epidemiological data reported elsewhere (36), thus likely excluding a strong impact of such a bias on the accuracy of GPs estimation. Furthermore, the short survey items were unable to explore in depth the opinions, knowledge, and clinical experience of the GPs. For example, asking about “positive” findings on neurological examination (e.g., reduction of symptoms with distractive maneuvers) would have helped to improve our understanding of GPs knowledge about the criteria actually used by neurologists to make a diagnosis of FND. Also, asking whether GPs use different management strategies with the same patients would have provided for a more in-depth understanding of their approach to FND in clinical practice.

## Conclusion

Our survey findings suggest that GPs need professional education about FND. Up-to-date guidance about appropriate terminology to use, mechanisms leading to FND, evidence-based treatments, and stages of the stepped care approach should be provided with the overarching aim of ensuring that patients receive consistent diagnosis, explanation, and management across primary and secondary care pathways. Steps in this direction have been undertaken by the study Authors, who have conducted specialist educational courses for GPs about the novel approach to FND and created an illustrative leaflet about recent advances in FND. These interventions need to be extended to a wider population of Italian GPs in order to increase the effectiveness of primary care for patients with FND.

## Data Availability Statement

The original contributions generated for this study are included in the article/Supplementary Materials, further inquiries can be directed to the corresponding author/s.

## Ethics Statement

The studies involving human participants were reviewed and approved by Ethics committee of the University of Verona. The patients/participants provided their written informed consent to participate in this study.

## Author Contributions

AM, MF, and MT: conception, design, organization of the research project, and interpretation of data. IF and CF: organization of the research project and acquisition of the data. AM: organization and acquisition of the data, design and execution of data analysis, writing the first draft and revision of it based on the other authors' critique. MF, IF, CF, GD, and MT: revised the work for critique. All authors approved the final version to be published.

## Conflict of Interest

The authors declare that the research was conducted in the absence of any commercial or financial relationships that could be construed as a potential conflict of interest.
