# Supplementary material for: Functional Neurological Disorders as Seen by a Cohort of General Practitioners in Northern Italy: Evidence From an Online Survey
Source: Front Neurol. 2021 Jan 25;12:583672. doi: 10.3389/fneur.2021.583672 (PMC7868405; doi:10.3389/fneur.2021.583672)
Supplement: Supplementary file 1 [file Table_1.DOCX]

**Supplementary Table 1.** Section 2: Opinion, knowledge, and clinical experience

| **Italian version** | | **English translation** |
| --- | --- | --- |
| **Secondo te, quali aspetti sono predittivi in una diagnosi di disturbo neurologico non organico?** | | **Which of the following are predictive of a diagnosis of a non-organic neurological disorder?** |
| Contenzioso legale associato alla condizione del paziente | | Litigation |
| Indagini neurologiche dettagliate rivelatesi normali o inconcludenti | | Normal or inconclusive neurological exams |
| Perdita di funzionalità o disabilità eccessiva rispetto a quanto emerso dagli esami medici | | Greater functional loss or disability than found on physical examination |
| Presenza di altri sintomi medicalmente non spiegabili | | Other medically unexplained symptoms |
| Remissioni spontanee verificatesi in passato | | History of spontaneous remissions |
| Storia pregressa di disturbi mentali o fattori di stress psicologico | | History of mental illness or psychological stress factors |
| **Secondo te, quanto sono indicati i seguenti trattamenti/figure specialistiche per un disturbo neurologico non organico?** | **In your opinion, how adequate are the following treatment/specialist consultations for neurological non-organic disorders?** | |
| Trattamento farmacologico per il tipo specifico di sintomo | Pharmacological treatment of symptoms | |
| Psicoterapia associata a antidepressivo o ansiolitico | Psychotherapy with antidepressant or anxiolytic medications | |
| Psicoterapia non associata a antidepressivo o ansiolitico | Psychotherapy without antidepressant or anxiolytic medications | |
| Servizi riabilitativi (es., fisioterapia, biofeedback) | Rehabilitation (e.g., biofeedback, physiotherapy) | |
| Intervento educativo | Educational intervention | |
| Visita psichiatrica | Psychiatric examination | |
| Visita neurologica | Neurological examination | |
| Visita fisioterapica | Physiotherapy examination | |
| Visita psicoterapeutica | Psychotherapy examination | |
| **Nel caso si presentasse nel tuo studio un paziente con il sospetto di sintomi neurologici non organici, cosa faresti?** | **If a patient with suspected non-organic neurological symptoms made an office visit, what would you do?** | |
| Prescriverei innanzitutto una visita neurologica | Write a referral to a neurologist | |
| Prescriverei innanzitutto una visita psichiatrica | Write a referral to a psychiatrist | |
| Prescriverei una visita da un altro specialista | Write a referral to another specialist | |
| Prescriverei delle indagini strumentali | Write an order for diagnostic tests | |
| Prescriverei dei famarci | Write a drug prescription | |
| Aspetterei di vedere come evolve il disturbo | Wait to see how symptoms develop | |
| **Nel caso si presentasse nel tuo studio un paziente con sintomi neurologici non organici, quale sarebbe il tuo grado di soddisfazione nel gestirlo?** | **What would be your level of satisfaction in managing a patient presenting with non-organic neurological symptoms?** | |
| [Indicare un numero da 0 (nessuna soddisfazione) a 10 (moltissima soddisfazione)] | [Select a number from 0 (no satisfaction) to 10 (high satisfaction)] | |
| **Quale ritieni possa essere il ruolo del Medico di Medicina Generale nella gestione dei pazienti con disturbi neurologici non organici? [Puoi indicare più di una risposta]** | **What is the general practitioner’s role in the management of patients with non-organic neurological disorders? [You can indicate more than one answer]** | |
| Porre la diagnosi e seguire il paziente personalmente | Make the diagnosis and personally follow-up the patient | |
| Porre la diagnosi e indicare al paziente la terapia più adatta al suo caso | Make the diagnosis and recommend the most appropriate treatment | |
| Inviare il paziente dallo specialista più adatto al suo caso | Refer the patient to the specialist most appropriate for the condition | |
| Seguire il processo di cura in collaborazione con il medico specialista che ha posto la diagnosi | Follow-up the treatment together with the specialist who made the diagnosis | |
| Attuare un intervento educativo di supporto al paziente e alla famiglia | Provide for education of the patient and family members | |
| Altro (specificare) | Other (please, specify) | |
