# Supplementary material for: Functional Neurological Disorders as Seen by a Cohort of General Practitioners in Northern Italy: Evidence From an Online Survey
Source: Front Neurol. 2021 Jan 25;12:583672. doi: 10.3389/fneur.2021.583672 (PMC7868405; doi:10.3389/fneur.2021.583672)
Supplement: Supplementary file 2 [file Table_2.DOCX]

**Supplementary Table 2.** Opinion on the predictive criteria for FND diagnosis. Responses – no.(%)

|  | I don’t know | Not predictive at all | Not very predictive | Somewhat predictive | Very predictive | Extremely predictive | Average Rating |
| --- | --- | --- | --- | --- | --- | --- | --- |
| Extensive normal or inconclusive neurological examinations | 0 (0) | 5 (4) | 8 (6) | 27 (20) | 54 (41) | 39 (29) | 3.86 |
| Previous mental illness or psychological stress | 1 (1) | 2 (2) | 12 (9) | 40 (30) | 46 (35) | 32 (24) | 3.71 |
| Greater loss of function or disability than found on physical examination | 3 (2) | 3 (2) | 14 (11) | 46 (35) | 52 (39) | 15 (11) | 3.48 |
| Spontaneous remissions | 3 (2) | 3 (2) | 21 (16) | 42 (32) | 49 (37) | 15 (11) | 3.40 |
| Other medically unexplained symptoms | 3 (2) | 3 (2) | 11 (8) | 57 (43) | 51 (38) | 8 (6) | 3.38 |
| Litigation | 17 (13) | 23 (17) | 21 (16) | 45 (34) | 20 (15) | 7 (5) | 2.72 |
| Factors are listed in descending order of rating average. | | | | | | | |
