# Supplementary material for: Functional Neurological Disorders as Seen by a Cohort of General Practitioners in Northern Italy: Evidence From an Online Survey
Source: Front Neurol. 2021 Jan 25;12:583672. doi: 10.3389/fneur.2021.583672 (PMC7868405; doi:10.3389/fneur.2021.583672)
Supplement: Supplementary file 3 [file Table_3.DOCX]

**Supplementary Table 3.** Opinion on specialist consultation and treatment adequacy for FND. Responses – no. (%)

|  | I don’t know | Not adequate at all | Not very adequate | Somewhat adequate | Very adequate | Extremely adequate | Average rating |
| --- | --- | --- | --- | --- | --- | --- | --- |
| **Specialist consultation** |  |  |  |  |  |  |  |
| Neurological consultation | 2 (1) | 2 (1) | 15 (11) | 39 (30) | 56 (42) | 19 (14) | 3.57 |
| Psychotherapy consultation | 2 (2) | 5 (4) | 22 (16) | 49 (37) | 37 (28) | 18 (13) | 3.31 |
| Psychiatric consultation | 0 (0) | 6 (5) | 28 (21) | 51 (38) | 40 (30) | 8 (6) | 3.12 |
| Physiotherapy consultation | 4 (3) | 7 (5) | 39 (29) | 49 (37) | 25 (19) | 9 (7) | 2.92 |
| **Treatment** |  |  |  |  |  |  |  |
| Rehabilitation (e.g., biofeedback, physiotherapy) | 10 (8) | 1 (1) | 22 (17) | 47 (35) | 33 (25) | 20 (15) | 3.40 |
| Educational interventions | 5 (4) | 3 (2) | 22 (16) | 45 (34) | 42 (32) | 16 (12) | 3.36 |
| Psychotherapy with antidepressant or anxiolytic medications | 3 (2) | 4 (3) | 21 (16) | 56 (42) | 42 (32) | 7 (5) | 3.22 |
| Psychotherapy without antidepressant or anxiolytic medications | 5 (4) | 2 (1) | 21(16) | 65 (49) | 35 (26) | 5 (4) | 3.16 |
| Pharmacological treatment | 0 (0) | 7 (5) | 26 (20) | 78 (59) | 19 (14) | 3 (2) | 2.89 |
| Factors are listed in descending order of average rating. | | | | | | | |
