# Supplementary material for: Functional Neurological Disorders as Seen by a Cohort of General Practitioners in Northern Italy: Evidence From an Online Survey
Source: Front Neurol. 2021 Jan 25;12:583672. doi: 10.3389/fneur.2021.583672 (PMC7868405; doi:10.3389/fneur.2021.583672)
Supplement: Supplementary file 4 [file Table_4.DOCX]

**Supplementary Table 4.** Management strategies. Responses - no. (%).

|  | Extremely disagree | Disagree | Uncertain | Agree | Extremely agree | Average rating |
| --- | --- | --- | --- | --- | --- | --- |
| Wait to see how symptoms will develop | 3 (2) | 24 (18) | 29 (22) | 61 (46) | 16 (12) | 3.47 |
| Referral to a neurologist | 6 (5) | 24 (18) | 24 (18) | 64 (48) | 15 (11) | 3.44 |
| Instrumental examination | 6 (5) | 21 (16) | 39 (29) | 60 (45) | 7 (5) | 3.31 |
| Referral to a psychiatrist | 8 (6) | 46 (35) | 44 (33) | 31 (23) | 4 (3) | 2.83 |
| Pharmacological prescription | 10 (7) | 42 (32) | 56 (42) | 24 (18) | 1 (1) | 2.73 |
| Referral to another specialist | 26 (20) | 71 (53) | 23 (17) | 11 (8) | 2 (2) | 2.19 |
| Factors are listed in descending order of rating average | | | | | | |
